# Cardiovascular Outcomes in Patients on Hemodialysis following Drug-Eluting versus Bare-Metal Coronary Stents

**DOI:** 10.1155/2018/4934982

**Published:** 2018-05-17

**Authors:** Amir F. Mohani, Srikanth Penumetsa, Amin Daoulah, Gregory Giugliano, Amir Lotfi

**Affiliations:** ^1^Department of Cardiovascular Medicine, Baystate Medical Center, University of Massachusetts Medical School, Springfield, MA 01199, USA; ^2^Deaconess Health System, Evansville, IN 47747, USA; ^3^King Faisal Specialist Hospital & Research Center, Jeddah 23736, Saudi Arabia

## Abstract

**Aim:**

This study sought to compare short- and long-term outcomes of drug-eluting stents (DESs) versus bare-metal stents (BMSs) implantation in patients with end-stage renal disease on hemodialysis (ESRD-HD) undergoing percutaneous coronary intervention (PCI).

**Methods:**

Adult patients with ESRD-HD who underwent PCI at all nonfederal hospitals in Massachusetts between July 1, 2003, and September 30, 2007, were stratified based on the stent type placed at index hospitalization: DES or BMS. The primary outcome compared was a composite of all-cause death, myocardial infarction (MI), congestive heart failure (CHF), target vessel revascularization (TVR), and stroke at 30 days and one year.

**Results:**

HD patients had a high mortality (31%) and were more likely to receive a DES than a BMS (77% versus 23%). Propensity score analysis of 2 : 1 matched DES (268) versus BMS (134) patients demonstrated the DES group to more likely have proximal LAD disease and a history of prior PCI. Conditional logistic regression analysis demonstrated no significant difference in the composite cardiovascular endpoint measured at 30 days (hazard ratio (HR) 1.09; 95% confidence interval (CI) 0.61–1.94) and one year (HR 1.03; 95% CI 0.68–1.57).

**Conclusions:**

There were no significant differences in 30-day or 1-year major cardiovascular outcomes in HD patients undergoing PCI using the DES compared to the BMS in this high-mortality patient cohort.

## 1. Introduction

The prevalence of patients with end-stage renal disease (ESRD) on hemodialysis (HD) is growing in the United States. As projected by disease trends, the estimated prevalence may have approached 700,000 patients by the year 2015 [1]. ESRD patients comprise 1% of total Medicare population and utilize approximately 7% of total Medicare expenditures, amounting to 32.8 billion US dollars annually [2]. Over the past three decades, crude mortality of patients on hemodialysis has decreased by about 26%, but it continues to be significantly higher, over 6.5–7.9 times as compared to the general population [2]. Their risk for mortality following a myocardial infarction (MI) and major adverse cardiovascular events (MACE generally includes all-cause mortality, nonfatal MI, congestive heart failure (CHF), stroke, and target vessel revascularization (TVR) following percutaneous coronary interventions (PCIs)) is much higher compared to nondialysis-dependent patients [3]. Multiple randomized studies have demonstrated the superiority of drug-eluting stents (DESs) over bare-metal stents (BMSs) in reducing both short- and long-term adverse cardiac events in patients with normal renal function [4–6]. However, patients with advanced renal failure and dialysis-dependent patients have generally been excluded from most of these randomized studies [7]. Hence, the benefit of the DES over the BMS in this group remains uncertain. In the absence of data from randomized studies, clinical decision-making has relied on retrospective or registry data to evaluate the performance of different stents in these high-risk patients, the results of which are equivocal at best and confounded due to the small sample size and methodological limitations [8–15].

## 2. Materials and Methods

### 2.1. Data Collection

The primary data source is assembled by the Massachusetts Data Analysis Center (Mass-DAC), a data-coordinating center established in 2002 in response to a state mandate to assess the quality of cardiac surgery and coronary interventions in all nonfederal hospitals located in the Commonwealth of Massachusetts. Trained data managers at hospitals collect patient-specific clinical data for all patients aged 18 or older at the time of their procedure. Mass-DAC utilizes the American College of Cardiology's National Cardiovascular Data Registry (NCDR) data collection instrument for PCI and the Society of Thoracic Surgeons' National Adult Cardiac Database instrument for cardiac surgery. The anonymized clinical data are securely sent to the Mass-DAC where a committee constituting physicians, surgeons, and clinical data statisticians adjudicates risk factors and outcomes.

Information on vital status for Mass-DAC subjects is obtained from the Massachusetts Registry of Vital Records and Statistics for Massachusetts's residents and from the Centers for Disease Control (CDC) National Death Index for subjects with a non-Massachusetts address. Hospital discharge information from the Division of Health Care Finance and Policy (now, the Center for Health Information Analysis) that contains sociodemographic information (age, sex, and race/ethnicity), health insurance information, and diagnostic and procedure codes for all subjects hospitalized in nonfederal hospitals located in the state is also utilized.

### 2.2. Patient Population

All adults (age > 18 years) on hemodialysis who underwent PCI at all acute-care nonfederal hospitals in the state of Massachusetts between 07/01/2003 and 09/30/2007 were identified, inclusive of all clinical presentations (acute coronary syndrome or elective).

### 2.3. Treatment Groups

Patients were classified as “DES treated” or “BMS treated” based on the stent type implanted at the index admission. Patients who had more than one stent type placed were eliminated from the analysis. Patients with multiple stents of the same stent type were included once and classified based on the index event/admission.

### 2.4. Outcomes

The primary outcome was a composite of all-cause death, MI, CHF, TVR, and stroke at 30 days and one year from the procedure. Secondary outcomes included the individual components of the primary outcomes.

Baseline demographic, clinical, and insurance data as well as lesion characteristics (NCDR defined) were derived from Mass-DAC reports. In-hospital mortality is directly reported to the Mass-DAC and confirmed by linkage with the Massachusetts Registry of Vital Records and Statistics, which also provides long-term mortality data. The Social Security Death Index and direct hospital inquiry were used to resolve discrepancies. MI data during index hospitalization were available as part of the Mass-DAC reporting tool and defined according to NCDR definitions. Subsequent events (components of the primary outcome) data were identified from hospital administrative and discharge diagnosis coding data. Events reported to the Mass-DAC in association with subsequent procedures or hospitalizations following the index admission were adjudicated by a panel of clinicians. Target vessel revascularization data, defined as any PCI performed on a vessel treated during the index procedure or any coronary artery bypass grafting (CABG) after the index procedure, were obtained from the Mass-DAC PCI Database, the Mass-DAC Cardiac Surgery Database, and hospital discharge billing data.

### 2.5. Statistical Analysis

Risk-adjusted mortality, MI, CHF, and TVR differences between DES and BMS groups were estimated with 2 : 1 propensity score matching and conditional logistic regression analyses, based on clinical, procedural, and insurance information collected at the index admission. A propensity score for receiving a drug-eluting stent was modeled as a function of the variables identified. The estimated log odds of the probability of a given patient receiving a drug-eluting stent (the logit) were calculated. Using the estimated logits, 2 DES patients were matched with one closest BMS patient using a caliper width of 0.6 of standard deviation. Fine balancing without the exact matching method was used. Continuous variables were reported as mean ± standard deviation, categorical variables were reported as percentages, and *χ*^2^ test was used for comparison. Conditional logistic regression analysis was performed to compare clinical outcomes between BMS and DES groups and presented with 95% confidence intervals (CIs). All statistical tests were 2-sided, and *P* < 0.05 was considered statistically significant. All statistical analyses were performed using SAS version 9.3 (SAS institute Inc.; http://www.sas.com).

## 3. Results

During the study period (07/01/03 to 09/30/07), a total of 44,253 patients underwent PCI with stent placement in Massachusetts. There were 593 hemodialysis patients in this cohort, constituting 1.34% of the total population. Patients on dialysis were more likely to receive a DES than a BMS (77% versus 23%). The overall mortality in the cohort was high at 31% at one year.

**Figure 1:**
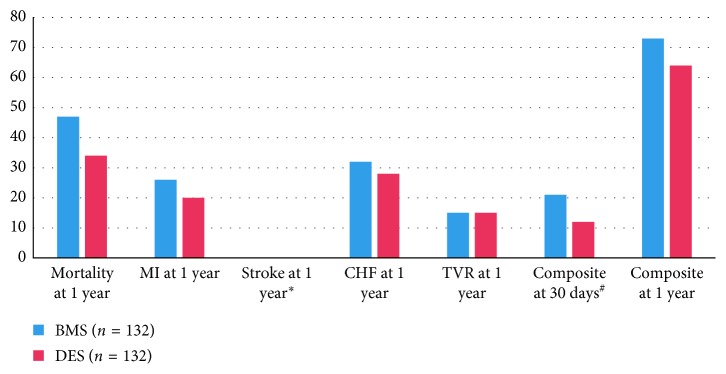
Comparative outcomes at 30 days and 1 year in BMS versus DES propensity-matched ESRD patients undergoing PCI. No statistical difference between groups was noted. ^∗^No stroke events were reported in either group. If there are fewer than 7 cases/events, the exact number is not disclosed per the Massachusetts Department of Public Health; hence, most individual 30-day events were suppressed; ^#^composite endpoint of the occurrence of any of the events (death, MI, stroke, HF, and TVR); BMS, bare-metal stent; DES, drug-eluting stent; ESRD, end-stage renal disease; PCI, percutaneous intervention; MI, myocardial infarction; CHF, congestive heart failure; TVR, target vessel revascularization.

Overall, the patients in both groups were a high-risk population with similar comorbidities (Table 1). Patients in the DES group were more likely to have proximal LAD disease and a history of prior PCI. The DES group was less likely to have a history of CHF. Propensity score analysis of 2 : 1 matched DES versus BMS patients was performed for 268 and 134 patients in the DES and BMS groups, respectively (Table 2). Based on conditional logistic regression analysis comparing the BMS versus DES, there was no significant difference in the composite endpoint of major adverse cardiovascular events at 30 days (hazard ratio 1.09, 95% CI 0.61–1.94) and one year (hazard ratio 1.03, 95% CI 0.68–1.57) (Table 3); the hazard ratios for mortality, MI, CHF, and TVR at 1 year were 1.13 (95% CI 0.72–1.77), 1.24 (95% CI 0.73–2.10), 1.20 (95% CI 0.72–1.98), and 1.17 (95% CI 0.60–2.27), respectively (Table 3). Individual and composite outcome comparison in 2 : 1 propensity-matched cohort did not show any significant differences at 30 days and 1 year between either stent groups (Figure 1). Information on anticoagulation during PCI was available for only 264 out of 593 (45%) patients in our study. Bivalirudin was used in 26% versus heparin in 74%. There was no statistically significant difference in bleeding or composite events between the two regimens (Table 4), although the power to detect any potential statistically significant differences between the anticoagulation regimens or between stent types was limited by the small sample size for which data on medication use were available.

## 4. Discussion

The results of this dedicated analysis of patients on dialysis undergoing PCI in the state of Massachusetts did not demonstrate any safety or efficacy differences between the DES and BMS.

Patients with end-stage renal disease on hemodialysis represent a unique population at significantly increased risk for cardiovascular disease. For those patients with known CAD, the need for dialysis conveys an increased risk of AMI, CHF, and sudden cardiac death, which may be realized within the first 6 months of initiating renal replacement therapy. Although cardiovascular mortality has decreased significantly among patients on hemodialysis in the past decade, mortality of Medicare patients on dialysis is ten times greater than that of age-matched patients without kidney disease. In the overall dialysis population, adjusted mortality rates are 6.5–7.9 times greater than those of the general population [2]. Cardiovascular risk factors and event rates are much higher in patients on dialysis, and only half of hemodialysis patients live past three years [16]. Moreover, CKD patients who underwent CABG or PCI had a higher risk of all-cause and cardiac mortality and increased cardiac admissions, with 70% patients with CKD and DM dead by 7 years [17]. In light of these sobering statistics, this patient population should be the subject of evaluation for impact of newer drugs and devices to improve outcomes. On the contrary, this high-risk subset of patients is typically excluded from clinical trials evaluating newer pharmaceutical or device therapies intended to reduce the risk of MACE. Furthermore, the limited studies available in ESRD patients of certain guideline-driven, standard of care therapies such as statins and AICDs for either primary or secondary prevention of cardiovascular events have had mixed results at best [18, 19].

Multiple large-scale randomized trials comparing the DES to the BMS have described the benefits of the DES over the BMS, including the reduction of restenosis and need for TVR. Some data suggest that DES use might reduce the incidence of myocardial infarction and death although conflicting evidence exists [8–15]. The high restenosis rates associated with BMS use in patients with chronic kidney disease and diabetes coupled with the dialysis population's high prevalence of CAD and diabetes (up to 80% in some cohorts) make restenosis prevention amongst dialysis patients particularly appealing. The lack of data on the DES in patients on dialysis reflects the exclusion of such patients from randomized controlled trials, necessitating interventionalists to make decisions regarding the stent type based on extrapolation of outcomes from trials that studied patients with only mild renal dysfunction. Recognizing the cost implications associated with DES compared with BMS, especially in the current medical-economic climate, the decision to use the most expensive device should be supported by proven efficacy. Data thus far have demonstrated the safety of the DES in dialysis patients, but the evidence regarding efficacy in reducing MACE remains scarce and even conflicting. The present analysis showed no significant clinical advantage of DES over BMS in dialysis patients. The high rates of clinical outcomes of 1-year mortality, TVR, restenosis, and MI in the study cohort are in concert with previously reported rates in this population, which should magnify the ability to detect any signal of benefit seen with DES [3, 20].

The study also confirms previously published data which show that only a small proportion of dialysis patients undergo PCI (regardless of the stent type), despite their high incidence of CAD. Perhaps, this reflects the high prevalence of diabetes and resultant multivessel disease in this population [21] and the current guidelines favoring CABG for such patients [22, 23]. Alternatively, physicians may be less aggressive in evaluating or intervening upon patients on dialysis for suspected CAD, or perhaps, these patients may exhibit silent ischemia. Lastly, it may be related to a presumption among treating physicians that there are few interventions that can substantially improve survival or quality of life among patients on dialysis. The increased risk of procedures coupled with equivocal and limited data regarding outcomes may tip the delicate risk/benefit ratio of PCI to be less favorable in dialysis patients than in the general population.

## 5. Study Limitations

First, as with all observational, retrospective analyses, our study is limited by potential unmeasured confounding for which adjustment could not be made despite our rigorous regression modeling and propensity-matched scoring analysis. Although propensity matching helps in controlling measured variables, some residual confounding persists from inherent biases in an observational cohort. Second, several variables that have important implications on outcomes in CAD patients were not accounted for. These include infarct territory (LAD versus others), infarct size, residual ischemic burden, and left ventricular hypertrophy; however, we attempted to thoroughly match the groups for clinical presentation, lesion complexity, CHF, and LVEF. It should be noted that, in the absence of a core laboratory, standardized data on lesion characteristics were obtained from participating hospitals and reported as such. Lesion data thus are limited without the availability of QCA data or any other systematic scoring method (e.g., SYNTAX score). Third, the Mass-DAC PCI database did not include the stent type/generation or concomitant medication use, including dual antiplatelet therapy duration, which may have impacted patient outcomes. The evolution of stent technology since, with availability of newer generation stents and pervasive use of newer antiplatelet drugs, limits extrapolation to contemporary clinical outcomes. The implications of choice of anticoagulant use during PCI (heparin versus bivalirudin), radial access utilization, vascular closure device use, or timing of achieving manual hemostasis for femoral access patients with respect to the anticoagulant used are all factors that may affect bleeding and ultimately overall clinical outcomes. Our data set is limited with respect to information on these variables. Moreover, the limited sample size also limits the external validity of the study findings. Given the lack of comparative outcomes data in patients with ESRD and CAD who were treated medically versus CABG, further studies are needed to determine the optimal treatment strategy for this high-risk population. Finally, our patient cohort comprised patients treated at nonfederal hospitals with mandated reporting in Massachusetts, and our results may not be generalizable to patients treated elsewhere in the country due to practice variations.

## 6. Conclusions

In summary, in this observational PCI database, there were no significant differences in 30-day or 1-year major cardiovascular outcomes in dialysis patients undergoing PCI using the DES compared to the BMS. Regardless of the type of the stent used, the overall mortality was significantly high in the dialysis patient cohort and demands dedicated randomized research.

## Figures and Tables

**Table 1:** Demographic, clinical, and procedural characteristics of patients before the propensity match.

Characteristic^∗^	BMS	DES	*P* value, DES versus BMS
	(*n*=134)	(*n*=459)	
Women	43 (32.09)	151 (32.90)	0.861
Age (years)	67.54 ± 11.90	66.26 ± 12.4	0.29
Race			0.128
White	110 (82.09)	360 (78.77)	
African American	8 (5.97)	47 (10.28)	
Hispanic	7 (5.22)	25 (5.47)	
Others	NA	18 (3.94)	
Insurance			0.244
Government	93 (69.40)	330 (71.90)	
Commercial	NA	60 (13.07)	
HMO	24 (17.91)	69 (15.03)	
No insurance	NA	0 (0.00)	
Past medical history			
Prior MI	53 (39.55)	218 (47.49)	0.105
CHF	58 (43.28)	182 (39.65)	0.452
Diabetes	77 (57.46)	306 (66.67)	0.05
Cerebrovascular disease	42 (31.34)	94 (20.48)	0.015
Peripheral vascular disease	54 (40.30)	192 (41.83)	0.752
Chronic lung disease	24 (17.91)	100 (21.79)	0.333
Hypertension	126 (94.03)	428 (93.25)	0.748
Dyslipidemia	111 (82.84)	362 (78.87)	0.315
Family history of CAD	32 (23.88)	132 (28.76)	0.268
Prior PCI	23 (17.16)	127 (27.67)	0.007
Prior CABG	40 (29.85)	115 (25.05)	0.267
Current smoker	18 (13.43)	56 (12.20)	0.705
Former smoker	73 (54.48)	242 (52.72)	0.721
Never smoked	43 (32.09)	161 (35.08)	0.523
Presentation			0.111
ST-elevation MI	17 (12.69)	30 (6.54)	
Non-ST-elevation MI	43 (32.09)	157 (34.20)	
Unstable angina	30 (22.39)	112 (24.40)	
Stable angina	11 (8.21)	54 (11.76)	
Noninvasive test outcome			0.44
No test	40 (29.85)	170 (37.04)	
Positive	74 (55.22)	228 (49.67)	
Negative	11 (8.21)	38 (8.28)	
Equivocal	9 (6.72)	23 (5.01)	
Congestive heart failure	58 (43.28)	143 (31.15)	0.009
NYHA classification			0.724
3	23 (17.16)	50 (10.89)	
4	25 (18.66)	57 (12.42)	
PCI status			0.298
Elective	44 (32.84)	171 (37.25)	
Urgent	69 (51.49)	241 (52.51)	
Emergency	20 (14.93)	46 (10.02)	
Left ventricular ejection fraction <30%	37 (27.61)	133 (28.98)	0.759
Number of vessels with lesions >50%			0.851
1	114 (85.07)	385 (83.88)	
2	19 (14.18)	68 (14.81)	
≥3	NA	NA	
Proximal LAD disease	16 (11.94)	87 (18.95)	0.038
Left main disease	7 (5.22)	20 (4.36)	0.673
High-risk lesion complexity	57 (42.54)	172 (37.47)	0.29
Number of vessels intervened on	1.16 ± 0.38	1.18 ± 0.42	0.589
Number of stents used, total	1.48 ± 0.83	1.64 ± 0.99	0.045
Per lesion	1.16 ± 0.58	1.16 ± 0.56	0.924
Per vessel	0.37 ± 0.21	0.41 ± 0.25	0.045
Periprocedural medications			
Thrombin inhibitors-bivalirudin	20 (25.64)	80 (30.42)	0.417
Heparin^†^	60 (44.78)	205 (44.66)	0.981
ASA	126 (94.03)	453 (98.69)	0.03
Clopidogrel	75 (55.97)	275 (59.91)	0.415
Glycoprotein IIb/IIIa inhibitor use	15 (11.19)	76 (16.56)	0.099

^∗^Continuous variables are expressed as mean ± standard deviation and categorical variables as number (percentage); BMS, bare-metal stent; DES, drug-eluting stent; MI, myocardial infarction; CHF, congestive heart failure; CAD, coronary artery disease; PCI, percutaneous coronary intervention; CABG, coronary artery bypass graft; NYHA, New York Heart Association; ^†^unfractionated and low molecular weight heparin.

**Table 2:** Characteristics of patients after 2 : 1 propensity match.

	BMS % (*n*=134)	DES % (*n*=268)	Standardized differences
Females	32.09	32.09	0.00
Age	67.54	66.91	0.05
Blacks	5.97	6.34	−1.55
Hispanics	5.22	5.22	0.00
CAD	23.88	25.75	−4.31
Previous MI	39.55	42.16	−5.30
CABG	29.85	32.46	−5.63
PCI	17.16	17.91	−1.96
CHF	43.28	42.16	2.26
CHF-NYHA IV	18.66	17.54	2.90
PVD	40.30	42.91	−5.29
HLD	82.84	80.22	6.72
HTN	94.03	95.52	−6.69
CVD	31.34	26.87	9.84
Diabetes	57.46	57.46	0.00
LVEF < 30%	27.61	27.99	−0.83
Nonsmokers	13.43	14.18	−2.16
High-risk lesion	42.54	41.05	3.02
Commercial insurance	11.94	11.94	0.00
HMO insurance	17.91	15.67	5.98
Stable angina	8.21	9.70	−5.21
Unstable angina	22.39	23.51	−2.65
NSTEMI	32.09	34.70	−5.52
STEMI	12.69	9.70	9.45
Emergency PCI	14.93	12.69	6.47

BMS, bare-metal stent; DES, drug-eluting stent; CAD, coronary artery disease; MI, myocardial infarction; CABG, coronary artery bypass graft; PCI, percutaneous coronary intervention; CHF, congestive heart failure; NYHA, New York Heart Association; PVD, peripheral vascular disease; HLD, hyperlipidemia; HTN, hypertension; CVD, cerebrovascular disease; LVEF, left ventricular ejection fraction; NSTEMI, non-ST-elevation myocardial infarction; STEMI, ST-elevation myocardial infarction.

**Table 3:** Outcomes at 30 days and 1 year in BMS versus DES patients: results of conditional logistic regression models.

	Hazard ratio^∗^	95% confidence limits
Mortality		
30 days	1.10	0.52, 2.33
One year	1.13	0.72, 1.77
MI		
30 days	1.13	0.48, 2.67
One year	1.24	0.74, 2.10
Stroke		
One year	1.00	0.25, 4.00
CHF		
30 days	0.60	0.17, 2.18
One year	1.20	0.72, 1.98
TVR		
30 days	0.43	0.04, 4.61
One year	1.17	0.60, 2.27
Composite		
30 days	1.09	0.61, 1.94
One year	1.03	0.68, 1.57

^∗^Bare-metal stents versus drug-eluting stents. Models were also adjusted for cardiovascular diseases. MI, myocardial infarction; CHF, congestive heart failure; TVR, target vessel revascularization.

**Table 4:** Outcomes at 30 days: bivalirudin versus heparin in patients with ESRD undergoing PCI.

	Bivalirudin (*n*=68)	UFH/LMWH only (*n*=196)	*P* value
	*n* (%)	*n* (%)	
Bleeding	NA^‡^	9 (5%)	0.95
Mortality	NA	19 (10%)	0.11
MI	NA	15 (8%)	0.94
Heart failure	NA	NA	NA
Stroke^†^	0 (0%)	0 (0%)	NA
TVR	0 (0%)	NA	NA
Composite^∗^^,†^	10 (15%)	35 (18%)	0.55
Composite BMS	2 (12/68)	8 (43/196)	0.88
Composite DES	8 (56/68)	27 (153/196)	0.57

^∗^Any of the following 30-day outcomes: mortality, MI, heart failure, stroke, or TVR; ^†^no stroke events were reported in either group; ^‡^if there are fewer than 7 cases/events, the exact number is not disclosed per the Massachusetts Department of Public Health; ESRD, end-stage renal disease; PCI, percutaneous coronary intervention; UFH, unfractionated heparin; LMWH, low molecular weight heparin; MI, myocardial infarction; TVR, target vessel revascularization; NA, not available; BMS, bare-metal stent; DES, drug-eluting stent.

## Abbreviations

BMS: Bare-metal stent
CABG: Coronary artery bypass graft
CDC: Centers for Disease Control
CHF: Congestive heart failure
CI: Confidence interval
DES: Drug-eluting stent
ESRD: End-stage renal disease
HD: Hemodialysis
MACE: Major adverse cardiovascular events
MI: Myocardial infarction
NCDR: National Cardiovascular Data Registry
PCI: Percutaneous coronary intervention
TVR: Target vessel revascularization.
